# The Experiences of Caregivers of Persons Living with Dementia in Jamaica during COVID-19

**DOI:** 10.1177/23337214211043384

**Published:** 2021-09-24

**Authors:** Marissa Stubbs, Ishtar Govia, Janelle N. Robinson, Rochelle Amour, Emily Freeman

**Affiliations:** 1 Epidemiology Research Unit, Caribbean Institute for Health Research, The University of the West Indies, 54657 Mona Campus, Kingston, Jamaica; 2 Care Policy Evaluation Centre, 4905 The London School of Economics and Political Science, London, UK

**Keywords:** Dementia, caregivers, Jamaica, COVID-19, thematic analysis

## Abstract

This article provides descriptive insights of the experiences of family caregivers of persons living with dementia during the COVID-19 pandemic. Data were generated as part of a qualitative cross-national project to explore the costs and consequences of providing unpaid dementia care. Participants in Jamaica, who were recruited using community gatekeepers, information booths at health fairs, conferences, and other outreach events, were contacted by telephone to discuss their experiences of the pandemic. When face-to-face in-depth interview data collection was suspended due to the pandemic, ethical approval was received to contact all research participants who were informal unpaid family caregivers, both those whose care recipients had died and those who were active caregivers (*N* = 19). Participants in this study were the 10 active family caregivers (*n*_*F*_ = 8; aged 45+; 60% from high socio-economic status). Their updates and reflections during these calls were documented in fieldnotes and analyzed for key themes. Data showed that the pandemic has illustrated the direct costs, both financial and otherwise, that informal dementia carers bear in Jamaica. It also intensified pre-existing challenges faced by family carers. We provide recommendations for sustainable support for family carers.

## Introduction

The COVID-19 pandemic has had unprecedented effects on the livelihood and health of people across the globe. However, people living with dementia (PLWD) and their carers stand to be disproportionately affected by both severe illnesses caused by the virus and the socio-economic repercussions of the pandemic (Giebel et al., 2020). On the one hand, as a vulnerable population, contact with others places them at risk for contraction of the disease. On the other hand, progression of dementia may be negatively influenced by measures and protocols that have reduced the availability of support systems and resources. This compromises informal carers’ ability to provide adequate care within an already underserved care context (Giebel et al., 2020).

In many low- and middle-income countries (LMICs) such as Jamaica, there are very few formalized care services for PLWD. In Jamaica, dementia care is typically provided informally in private households. This is constituted of unpaid –mostly female– family members, with or without assistance from others in the informal care sector, specifically those who are paid in cash and/or lack formal employment contracts (CaPRI, 2018; Govia et al., 2021). This results in excessive costs to family carersdirect and indirect financial costs, relationship costs, and negative health outcomes (Ferri & Jacob, 2017; Prince et al., 2009; Wimo et al., 2017). During the pandemic, these carers have had to navigate government-mandated lockdowns and curfews, restricted public social gatherings, and stay-at-home orders for older persons (Amour et al., 2020; Office of the Prime Minister, 2021). These mandates, which have been enforced by law, were enacted in efforts to control the spread of the novel COVID-19 virus and protect older adults.

The costs, consequences, and realities of providing unpaid care for PLWD in LMIC contexts like Jamaica is under-researched (Ferri & Jacob, 2017; Prince et al., 2009; Wimo et al., 2017), and there is a further paucity of evidence about the potentially complex realities of caregivers in LMIC contexts during a crisis (Rajagopalan et al., 2021). Such evidence is crucially important for developing appropriate responses and management strategies that are sensitive to the needs of both PLWD and their caregivers. This article presents experiences and challenges faced by family caregivers of PLWD in Jamaica during the COVID-19 pandemic.

## Methods

Qualitative data in Jamaica were collected as part of a cross-national project about the costs and consequences of providing unpaid dementia care (other countries were India and Mexico). The project was conducted by two experienced qualitative researchers and a team of more novice qualitative researchers who received in-depth training as part of the larger project. We used a theoretical sampling strategy designed to maximize variation in key social and demographic characteristics likely to influence caregiving experiences (see Table 1). We aimed for a stratified sample by socio-economic status (SES) classifications that were based on the Social Development Commission’s (2021) community classifications and location in rural and urban areas (as defined by the Statistical Institute of Jamaica). Recruitment, carried out between October 2019 and March 2020 in the parishes of Kingston and St. Andrew, involved the use of gatekeepers in communities identified for different target SES categories, and one-on-one engagement with interested persons at health fairs, conferences, and other outreach events.

**Table 1.** *Characteristics of Unpaid Family Caregivers* (*n* = 10) and Care Recipients (*n*=10).

Characteristics	Frequency	
Age of caregiver	*N*	%
45–55	5	50
55–64	3	30
65+	2	20
Sex of caregiver		
Male	2	20
Female	8	80
SES of caregiver		
High	6	60
Middle	2	20
Low	2	20
Age of care recipient		
60–75	2	20
76–89	7	70
90+	1	10
Sex of care recipient		
Male	2	20
Female	8	80
Care recipient dementia stage		
Moderate	9	90
Severe	1	10
Care recipients’ relationships to caregiver		
Father	3	30
Mother	5	50
Spouse/partner	2	20
Care recipients’ setting		
Caregiver’s home	4	40
PLWD’s home	4	40
Nursing home	2	20
Use of additional formal/paid care services		
Yes	9	90
No	1	10

Data collected during October 2019 and March 2020 (*n* = 19) included both active family carers of PLWD (*n* = 10) and former family carers of PLWD (*n* = 9). Following the onset of the COVID-19 pandemic in Jamaica in March 2020, ethical approval was received from the London School of Economics and Political Science [REC ref. 000834] in the UK and the University of the West Indies, Mona, Jamaica [ECP 122, 18/19 to contact all participants throughout the pandemic either via telephone or WhatsApp Messenger, depending on the individual participant’s preference. We developed and used open-ended scripts to guide exchanges via telephone and texts (approximately five times per participant, between April 2020 and May 2021). Participants were prompted to share their experiences adjusting to providing care during the pandemic at the time of contact and, based on support needs identified, were made aware of any community support resources available. Interactions ranged between 2–30 minutes, depending on how much the participant was willing to share. We typed detailed summaries of these conversations into a securely stored spreadsheet and conducted inductive thematic content analysis of those summaries using QSR International’s latest NVivo qualitative data management software (Braun & Clarke, 2006; QSR International Pty Ltd, 2020).

The present study focuses on participants who were active family carers (*n* = 10) at the beginning of the pandemic (see Table 1). We learned from the calls that three of the persons living with dementia died during the pandemic.

## Results

We identified four broad themes in the data and below provide details on these descriptive insights of the experiences of unpaid family caregivers: accessing support services, adapting to protocols, changes to care system and impact on carer wellbeing, work, and financial status.

### Theme 1: Accessing support services

Participants’ detailed barriers they experienced in using key services and their strategies to overcome these. Two main sub-themes were: *impact of limited availability of medical support* and *responses to limited services.*

*Impact of limited availability of medical support.* When managing other chronic comorbid conditions experienced by the PLWD, three participants reported that the lack of timely medical care exacerbated the severity of initial medical concerns and subsequently increased the cost of care. For example, one participant providing care at the PLWD’s residence, with assistance from paid carers, described how, unable to secure at-home medical care, she had to use costly ambulance services to take her husband to the hospital when he was on the brink of renal failure. While another, who was also providing care at the PLWD’s residence but lacked sufficient resources to use paid care or access at-home medical care, described the exorbitant hospital fees she had to pay for her father to be admitted when his swollen foot became infected due to inaccessibility of immediate, appropriate care from a free clinic when needed.

While seven participants reported using dementia medication to manage symptoms, only one participant, who provided care in her home for her mother with moderate dementia symptoms, expressed concerns about sourcing medication locally due to manufacturers abroad downsizing and reducing shipments.

*Responses to limited services.* Formal dementia carer support services are minimal in Jamaica, but participants noted that before the pandemic more general psycho-social supports were available, provided by church groups and non-governmental organizations, geared towards stress management (Govia et al., 2021). Following the shift to remote service provision, eight participants from the middle to upper SES classification, noted that they were able to continue to access social supports virtually or, more importantly, access new supports they could not access pre-pandemic due to the in-person time commitments or their immobility. For one participant in the lower SES classification, however, this switch to virtual platforms was a barrier to accessing supports, like church groups, that they had used prior to the pandemic.

Six participants across all three SES groups described how word of mouth was crucial in accessing dementia-related information and resources during the pandemic, such as guidance on effective dementia care strategies, paid carer recommendations, and appropriate equipment and materials. These participants also highlighted their ability to act as both sharer and receiver of such resources in their social networks.

### Theme 2: Adapting to protocols

Given widespread knowledge about the increased susceptibility and vulnerability of older persons to COVID-19, this theme captured the experience of unpaid carers as they attempted to navigate protocols’ impact on everyday life. Four sub-themes were identified: *enforcing mitigation strategies, managing loss,* *restricting* *in-person/physical gatherings*, and *transportation challenges.*

*Enforcing mitigation strategies.* Most participants spoke of adapting and enforcing the government-mandated mitigation strategies because they recognized that doing so was ensuring the safety of the person for whom they were providing care. All participants highlighted their consistent use of face coverings, sanitization methods, and immune boosting strategies, such as increased attention to nutrition. Two participants, both over the age of 60, noted that these strategies were also useful in protecting themselves from infection.

*Managing loss.* Four participants reported loss since the onset of the pandemic. While COVID-19 was not the cause of death, three participants reported the death of their care recipient and one reported loss of another family member.

One participant noted that reduced visitations at residential homes and the hospital decreased opportunities to see her father before his death. Family dynamics also impacted the grieving process. One participant detailed her frustration in delaying memorial ceremonies because her siblings residing outside Jamaica did not want their father to be buried in their absence. The participant that lost a family member who was not the PLWD care recipient, spoke of the difficulty of managing the care of a PLWD during funeral ceremonies when family members attempted to circumvent physical distancing restrictions and pressured the participant to include the PLWD in related activities.

*Restricting in-person/physical gatherings.* Two participants from the high SES classification described how in-person activities had been a regular part of their care routine. A participant who provided care in her home for her mother, a wheelchair user, noted that taking her mother to the salon to wash her hair used to be an activity her mother really enjoyed and that it was difficult trying to explain to her mother why she could no longer go to the salon. Another participant, whose father was living with dementia at a nursing home, shared that both she and her mother were sad and frustrated because the ongoing visitation restrictions meant that they were unable to visit him.

Four participants from middle and low SES classifications who provided care within their homes expressed concern about the extended lack of social engagement and stimulation for the PLWD. Two of them said that they felt personally responsible for ensuring the health and safety of their homes and that the restrictions were necessary to reduce their own and their care recipient’s risk of contracting the virus. They also discussed how increased vulnerability of PLWD significantly reduced their own social engagement. One participant, co-residing with the PLWD, spoke about no longer allowing guests to visit their home, while another spoke about how quiet the house has become since they were no longer inviting friends over.

*Transportation challenges.* Some participants noted that transportation during the pandemic contributes significantly to either increasing or reducing the risk of exposure to the virus. Two participants from the high SES classification who owned vehicles shared that limiting running of errands for the PLWD in turn limited both their and PLWD’s risk of catching the virus. However, two other participants who had paid carers that relied on public transportation, expressed concern about potential risk of virus exposure to the PLWD. One participant, who herself used public transportation shared her fear and guilt of potentially bringing the virus home.

### Theme 3: Changes to care system

The unprecedented nature of the pandemic led many carers to adapt their care arrangements to maintain quality care and a good quality of life for the PLWD. Four sub-themes were identified: *efforts to improve mobility, increased support needs, maintaining social connection*, and *modifying care.*

*Efforts to improve mobility.* With ongoing restrictions on mobility, participants who provided care at their home described attempts to create active outlets for the PLWD. These efforts were particularly important for one participant who cared for an immobile PLWD since the stay-at-home order for older persons reduced opportunities for PLWD to venture into the public. Two participants described more intentional efforts to provide minor changes in scenery, for example, by moving their care recipient to sit outside to get fresh air or taking the PLWD on drives.

*Increased support needs.* Given the progressive nature of dementia, eight participants reported increased care needs, for example, first-time incontinence, challenges with feeding, worsening of comorbid conditions, and difficulties moving persons who were immobile, during a time of restricted access to medical interventions and paid care options. While nine of the participants had utilized paid care, three of them who wanted to change or increase assistance acknowledged that hiring new or additional care workers could mean exposing the PLWD to the virus. Considering restricted access to free community medical resources, one participant who was providing care at home reported a desire to hire care workers with medical training (e.g., to help with prevention and treatment of bedsores) despite the significant cost implications.

*Maintaining social connection.* Considering concerns surrounding lack of engagement for the PLWD, three participants shared strategies such as regular telephone audio calls between the PLWD and close friends or relatives to ensure that the PLWD regularly had new conversations or virtual attendance at previously enjoyed activities, such as church, so the PLWD could have social interaction and stimulation.

As the pandemic progressed, both participants caring for their spouse shared that their offspring, who all lived abroad, paid them visits. Participants carefully planned these visits and ensured that they maintained all the necessary protocols. They also shared how meaningful these visits were for both the PLWD and themselves as carers. A female carer providing care for her husband described how, despite him being mostly non-verbal, her spouse vocalized to their child “I am so happy to see you” and “You must know how much you mean to me.”

*Modifying care.* One participant from the high SES classification, who used paid care services, discussed moving the PLWD into a part of the house that had less person-traffic. She noted that navigating all the persons in the house, including grandchildren who now had school at home, has been a challenging adjustment. The benefits of having multi-room homes were also evident with another high SES participant, an essential worker, who contracted COVID-19 and reported having to quickly convert a storage room in his home to a quarantine space to keep his mother who is living with dementia safe.

Though not prompted by COVID, a participant from a lower SES classification, noted how she moved her grandmother from Kingston to her grandmother’s countryside home in another parish, with a paid care worker previously known to the family, to reduce tension between her aunt and her grandmother. Ongoing COVID-19 movement restrictions coupled with lack of internet connection and phone services in the countryside parish, made it difficult to maintain regular contact with her grandmother after this move.

### Theme 4: Impact on carer wellbeing, work, and financial status.

As the primary caregivers of PLWD, participants’ wellbeing and financial status were instrumental to their ability to provide appropriate care. The following sub-themes capture the ways in which the pandemic has impacted participants’ means of providing unpaid care: *coping strategies*, *impact on employment*, and *financial strain.*

*Coping strategies.* At each point of contact, each of the participants expressed concerns around uncertainty, worrying about being alone, and fear of their family members catching the virus. Importantly though, they also spoke about coping strategies including spirituality, gardening, and exercise, noting that these resources offered them a sense of purpose as they continued to navigate the pandemic within their care roles.

*Impact on employment.* The pandemic had different effects on participants’ employment status and by extension their source(s) of income. Two participants who provided care at home noted that their place of employment had embraced remote work at various points during the pandemic which allowed them to spend more time caring for their loved one. For others, however, lack of flexibility to work remotely, as was the case for one participant who identified as an essential worker, often meant having to leave their homes to be at the physical workplace. Another participant noted that her working fewer hours meant that she did not get paid as much as before the pandemic.

*Financial strain.* Amidst increased support needs, four participants from the middle and low SES classification groups shared experiences of financial strain when attempting to fill the resource gaps in their care arrangements. As one participant noted: “...the biggest burden is the financials but other than that things are good…” (male carer providing care for his wife). Hospital fees and increased costs for paid care were two common expenses among this sub-group of carers. Two participants who provided care in their homes noted that they had to hire additional domestic workers to help with care provision. Because the pandemic negatively affected their own incomes, this need for additional help meant that they had to seek financial assistance from other family members.

## Discussion

Thematic analyses indicated that the pandemic has illustrated the direct costs (financial and otherwise) that informal dementia carers bear in Jamaica. These direct costs existed before COVID and persist during the pandemic despite differences in care setting, SES classification and use of additional formal/paid care services.

Direct costs during the pandemic included medical bills for the PLWD and hiring additional paid care workers, both with and without medical qualifications, as dementia symptoms progressed. Inaccessibility to timely medical support services and lack of inclusion of carers of PLWD in protocol considerations (e.g., carers were initially not exempt from travel restrictions during curfews) led to instances where participants' family members became ill and were left untreated, or participants attempted to fill the gap by hiring private nurses. This represented additional financial costs for participants, who were already shouldering significant costs of care, amidst a pandemic-induced atmosphere of increased job uncertainty and pay cuts.

Before the onset of the COVID-19 pandemic, unpaid carers constructed tailored, informal care systems with the resources available to them. In the context of Jamaica, these care systems existed despite a lack of dementia-specific social safety nets (Govia et al., 2021). While the construction of these informal care systems is commendable, the pandemic has emphasized their fragility. This has been particularly so for those carers without adequate resources to navigate the acute and chronic changes brought on by events like the pandemic. While none of the participants reported an inability to continue care, their resilience was likely made possible by privileges such as living in multi-room homes to facilitate social distancing in inter-generational homes, private vehicles, outdoor gardens to allow for changes of scenery, being able to afford additional paid in-home assistance and medical care costs, and internet access to access existing social support group networks. This suggests that for those without these resources, both quality of care and life for the PLWD may be negatively affected.

The restrictions in social engagement during the pandemic have also intensified the need for increased targeted social support for unpaid carers and for PLWD. While participants highlighted the role of their faith and personal social networks during the pandemic, they noted a significant gap in more targeted support options offered to carers of PLWD and for the PLWD themselves. Considering participants' identification of their faith as a coping mechanism, partnerships with places of worship-led support groups to support the needs of caregivers and persons living with dementia may be a useful intervention to formalize existing support available. However, given that some participants expressed challenges accessing previously available support, it will be important to provide diverse modalities of this resource such as offering recordings of any educational sessions asynchronously, sharing content via messaging groups (e.g., WhatsApp groups) and willingness to allow for physical delivery of any educational or psycho-social support resources.

As Jamaica undergoes population aging and international experts advocate for the need for better management of NCDs in LMICs, especially during the pandemic (Yadav et al., 2020), these experiences raise questions around the country’s preparedness, particularly in cases where informal carers are themselves older persons, such as spouses, siblings, or church brothers and sisters (church members with whom they have a close relationship bond). With the spotlight on older persons as a vulnerable group during the pandemic, this represents a unique opportunity to advocate for those seeking more targeted financial support as an informal carer of a person living with dementia. For example, lobbying for interventions to improve the sustainability of unpaid care for PLWD, especially during the COVID-19 pandemic, such as care subsidies to aid in costs of hiring trained care and subsidizing medical expenses, may be well-timed.

## Conclusion

The pandemic has illustrated the direct costs (financial and otherwise) that informal dementia carers bear in Jamaica. The findings suggest that increased care demands amidst diminishing care resources increases the vulnerability of both persons living with dementia and caregivers. While the body of research is growing about the experience of caregivers in the context of developing countries, continued engagement with informal carers is required to influence policy, inform interventions and guide resource allocation for future crises such as natural disasters, to which the region is vulnerable. Without appropriate social safeguards and assistance, persons face increased vulnerability during crises. While these informal care systems have thus far endured throughout the pandemic, this continuity comes at great costs to carers—extensive demands on finances, time, and emotional wellbeing—and with limited options for relief. Governmental organizations, advocacy groups, and places of worship have rallied to provide some support for older adults during the pandemic. However, it is essential to develop evidence-informed, dementia-specific resources and support to ensure that PLWD and their carers from all SES backgrounds live well and do not shoulder excessive burdens.
